# Identifying barriers to mental health system improvements: an examination of community participation in assertive community treatment programs

**DOI:** 10.1186/1752-4458-5-27

**Published:** 2011-11-07

**Authors:** Patricia A Wakefield, Glen E Randall, David A Richards

**Affiliations:** 1 Health Services Management, DeGroote School of Business, McMaster University, 1280 Main Street West, Hamilton, ON, L8S4M4, Canada; 2 Faculty of Business Administration, Lakehead University, 955 Oliver Road, Thunder Bay, ON P7B5E1, Canada

**Keywords:** health policy, peer support, fidelity, mental health system improvement, evidence-based practice, Ontario

## Abstract

**Background:**

Integrating the best available evidence into program standards is essential if system-wide improvements in the delivery of community-based mental health services are to be achieved. Since the beginning of the Assertive Community Treatment (ACT) program movement, program standards have included a role for the community. In particular, ACT program standards have sought to ensure that members of the local community are involved in governance and that former clients participate in service delivery as "Peer Support Specialists". This paper reports on the extent to which ACT program standards related to community participation have been implemented and identifies barriers to full compliance.

**Methods:**

Qualitative and quantitative data were collected through a telephone survey of ACT Program Coordinators in Ontario, Canada, using a census sample of the existing 66 ACT programs. A thematic approach to content analysis was used to analyze respondents' qualitative comments. Quantitative data were analyzed using SPSS 16.0 and included means, frequencies, independent t-tests and Pearson Correlations.

**Results:**

An 85% response rate was achieved. Of the 33 program standards, the two that received the lowest perceived compliance ratings were the two standards directly concerning community participation. Specifically, the standard to have a functioning Community Advisory Body and the standard requiring the inclusion of a Peer Support Specialist. The three major themes that emerged from the survey data with respect to the barriers to fully implementing the Community Advisory Body were: external issues; standard related issues; and, organizational/structural related issues. The three major themes concerning barriers to implementing the Peer Support Specialist role were: human resource related issues; organizational/structural related issues; and, standard related issues.

**Conclusions:**

The reasons for low compliance of ACT programs with community participation standards are complex and are tied to structural and human resources barriers (both internal and external to the ACT programs) as well as to the requirements of the standards themselves. In order for improvements to the mental health system to be achieved there is a need to identify and address these barriers. Failure to do so will result in less than optimal client, family and economic efficiency outcomes.

## Background

Integrating the best available evidence into program standards and practice is essential if system-wide improvements in the delivery of community-based mental health services are to be achieved. In the case of Assertive Community Treatment (ACT) programs, a second iteration of program standards has recently been developed in Ontario, Canada to facilitate the evidence-based delivery of these programs which are designed to provide community-based services to individuals with serious, persistent mental illness. Features of the ACT approach include: delivery of comprehensive services in individuals' immediate communities and homes; individualized treatment plans; access to services 24 hours a day, seven days a week; and, services provided by a collaborative team of multi-disciplinary experts. ACT program standards are developed to help ensure that treatment programs are implemented in a manner consistent with evidence-based practice. These standards typically include criteria for such functions as: organizational structure and communications; required staffing; procedures for intake, admission and discharge; maintenence of client records; clinical services; and community participation.

At the core of the ACT model's "community-based" philosophy is not only the delivery of services in the community but also the participation of members of the community in the governance of the program and the delivery of services. The use of community members in providing oversight to program operations (both as members of the Board of Directors and in an advisory capacity) has been a long-standing practice for organizations delivering community-based services, however, the use of "consumer providers" in the delivery of services, or "peer support specialists" as they are referred to in ACT programs, is a relatively recent phenomenon. Community participation activities have been identified as important elements in facilitating mental health recovery [1-4] which is at the core of their inclusion in formal program standards in other jurisdictions [5,6]. However, the importance of community participation standards does not appear to be reflected in either the academic research or programmatic evaluations of the ACT model since this area has been understudied and there is inadequate guidance for program administrators regarding how to successfully implement these standards. Thus, there is a need to identify and better understand how to overcome, barriers to the implementation of these community participation program standards to achieve system-wide improvements.

The purpose of this article is to report on the extent to which ACT programs in Ontario, Canada have implemented program standards relating to community participation and to examine specific barriers which may be inhibiting full compliance with these standards. We specifically examine the use of community advisory bodies (CAB) and the requirement that a peer support specialist be a member of each ACT team. For both of these standards we identify, from the Program Coordinator's perspective, the perceived levels of compliance with these standards, and how essential they believe these standards are to the effective functioning of their ACT program. This article begins with some background on the ACT model, its formal adoption in Ontario, and the two program standards that most directly relate to community participation - *Community Advisory Bodies *and *Peer Support Specialists*. The article then outlines the study's methodology and results, followed by an analysis and discussion of the major barrriers to implementation of the two community participation standards.

### Mental Health Reform

Mental health reform has been a high priority on the public policy agenda of most Ontario governments since the 1980s as is evident by the array of government reports produced [7-9]. Much like other health care reform initiatives, mental health reform has been driven by the need to control costs and the desire to repair deficiencies in the system. These deficiencies include fragmented availability of mental health services across the province, lack of accountability to clients and the public, and an inadequate ability to respond to local needs due to the government's centralized decision-making process [9].

Over the years, a major focus of mental health reform initiatives worldwide has been, and continues to be, a shift from institutional to community-based services. To facilitate this shift in Ontario, in the late 1990s the Ontario government began to divest itself of provincially owned psychiatric hospitals. As an alternative, the government directed more funds towards community-based care. By 1998 the Ontario government formally endorsed the use of ACT programs as a mechanism to reduce acute hospitalizations and support the provision of services to individuals with serious mental illness in the community [10].

ACT programs in Ontario are intended not only to provide community-based care but are to be sponsored by an organization within the immediate community. A community organization, usually a hospital or a not-for-profit health care organization, identifies the need for an ACT program within its community and applies to the Ministry of Health and Long Term Care for funding approval to operate an ACT program. If Ministry approval is granted, funds for the operation of the ACT program are administered through the sponsoring agency which is responsible for the operation of the program in accordance with program standards.

The approach on which the ACT program model is based originated in the United States in the early 1970s [11]. This community-based approach is predicated on the belief that serious and persistent mental illness requires intensive psychiatric, medical, and social support interventions and that these services are best provided in the community where the individual lives and must function, versus the traditional "institutionalized" approach which has been shown to be less effective [12,13].

Several measurment scales have been developed as a means to assess fidelity to ACT program standards [14-16]. The ACT model of care has long been shown to result in improved outcomes for individuals with serious mental illness [12] and, increased cost effectiveness versus other approaches to the delivery of mental health services [17,18]. Positive outcomes include: decreased family burden; reduced symptoms and program dropout rates; improved social functioning; reduced hospitalization rates; and, enhanced family satisfaction [17-23]. However, in order for these positive outcomes to be realized it is necessary for consistency in implementation of program standards. This evidence-based practice, linking positive outcomes to compliance with ACT program standards, encouraged the Ministry of Health and Long Term Care in Ontario to conduct a review of the 1998 ACT program standards. In 2004 the government issued a 39-page revised version of the Ontario Program Standards for ACT Teams [24], providing minimum standards for program operation and descriptions of the rationale for the requirements. The standards define:

*(1) for whom a program is intended; (2) the required services; (3) the type of staff/numbers needed to competently provide the services; and, 4) the intended benefits/outcomes for the clients receiving the services. Program standards are used to establish costs and are used for program monitoring and compliance purposes *[24].

Descriptions are provided for standards that include: intake, admission and discharge; service intensity and capacity; staff requirements; program organization and communication; client-centered assessment and individualized treatment and service planning; required services; maintenance of client records, procedures to resolve complaint resolutions; performance improvement and program evaluation; community advisory bodies; and accountability [24].

Two of the standards specfically provide for the inclusion of individuals from the immediate community in the functioning of the local ACT program. These are the standards for *Community Advisory Bodies *and for *Peer Support Specialists*.

#### Community Advisory Bodies

Under the 2004 program standards, each ACT program is to establish a Community Advisory Body (CAB) which consists of a group of volunteers from the local community, including consumers of mental health services, who provide advice to each ACT program. The specific standard notes that:

*The ACT team shall relate to a community advisory body which supports and guides ACT team implementation and operation ... The community advisory body is accountable and reports directly to the Board of Directors of the sponsoring agency. Members are chosen for their expertise in mental health or addiction services, their links with other relevant community services, their ability to represent the interests of clients and their families and the community, and other expertise required to direct a mental health service. Members should include mental health consumers and commuity stakeholders that interact with persons with serious mental illness ... [and] shall also reflect the diversity of the local population *[24].

While CABs do not have any formal authority over the operation of their ACT programs, previous research found evidence that the direct participation of qualified individuals and organizations from the community in the design and delivery of mental health services has a valuable impact on the success of the programs. For example, research findings suggest that the community's participation in mental health initiatives provides a valuable and different perspective from that of traditional health service providers [25] and, is an important factor in predicting healthier communities [26]. Thus, the program standard for CABs specifically requires that "... the community advisory body shall have written terms of reference incorporating the requirements outlined in ..." the revised ACT program standards [24].

#### Peer Support Specialists

The 2004 program standards also provide detailed requirments for staffing programs. Each ACT team is to be composed of specified minimum staffing levels, including a Program Coordinator, registered nurses, an occupational therapist, a substance abuse specialist, a vocational specialist, a peer specialist, other clinical staff (e.g., a psychiatrist), and a program/administrative assistant. The requirement for peer specialists, a unique addition to the traditional team of health professionals, pre-dates the revised 2004 standards for ACT teams in Ontario, although prior research indicates that there was considerable inconsistency with how the role was defined and its degree of integration with other ACT team members [27].

*ACT Teams are expected to promote client-centered practices by the deployment of a peer specialist .... Peer support services serve to validate clients' experiences and to guide and encourage clients to take responsibility for and actively participate in their own recovery. In addition, services help clients identify, understand, and combat stigma and discrimination against mental illness and develop strategies to reduce self-imposed stigma *[24].

The Peer Support Specialist is a representative from the community who has had personal experience in treatment program(s) for serious mental illness, and who can provide counselling and support to current clients. Services provided by the Peer Support Specialist include: serving as a role model; helping clients to develop coping mechanisms; sharing experiences; educating the ACT team members and staff about the client perspective; serving as an advocate for the development of initiatives within the community that will facilitate client empowerment; and, making clients aware of self-help programs and organizations that can be helpful in their recovery [24].

The program standards require the use of Peer Support Specialists because they are believed to enhance the overall functioning of ACT programs. Reported findings from several researchers indicate that Peer Support Specialists contribute to improved client/consumer outcomes when incorporated into the delivery of mental health services [28-31]. Despite this, the integration of non-professional peer service providers (or Peer Support Specialists) into the ACT service team is not well understood. This may be due, in part, to differences in role definition across programs and a paucity of rigorous empirical evaluation [32]. Mowbray et al. [29] found significant role confusion in some programs, where the peer support specialist may provide functions that serve as both friend and clinician; while Lyons et al. [33] identified challenges in implementation where Peer Support Specialists were less likely to be dispatched in an emergency than were other ACT team members. In their 2003 survey of ACT programs in Ontario, White et al. [27] found that Peer Support Specialists were not fully integrated into existing ACT teams; and, 22% of the respondents indicated that they were not planning to implement such a position. Thus, a number of challenges exist regarding the use and implementation of consumers as service providers in mental health services.

This study adds to the international literature on the integration of local community members in the design and delivery of mental health services. These findings will be instructive to an international audience in that they provide both quantitative and qualitative data generating new insights into barriers and facilitators to the compliance of program standards focused on enhancing the community's role in the provision of mental health services. Additionally, this study provides concrete information that may be used by decision-makers within Ontario, including politicians, sponsoring agencies, and program managers, to take decisive action to make improvements to the quality of local mental health programs and the services they deliver.

## Methods

This mixed methods study obtained both quantitative and qualitative data collected through the administration of a telephone survey.

### Participants and Data Collection

Eligible participants were ACT Program Coordinators (or their equivalent) for all 66 ACT programs in existence in Ontario at the time of data collection. A census sample of all of the current ACT Program Coordinators, as identified in a list obtained from the Ontario ACT Association, was used.

Ethics approval for this study was obtained from the McMaster University Ethics Review Board and the research was funded by a grant from the McMaster University Arts Research Board. Each survey respondent was required to sign a detailed consent form prior to participating in the study. Information about the study was originally provided to ACT Program Coordinators by email. Program Coordinators were then contacted by telephone to answer any questions they may have about the study and to arrange a future date and time to administer the telephone survey.

### Measurement Scale Development

The 2004 Ontario Program Standards for ACT Teams document is a 39-page report developed by the Standards Sub-Commitee of the ACT Technical Advisory Panel between September 2003 and June 2004 [24]. Its content is based on the American standards document, *The National Program Standards for ACT Teams *[34], which was written to serve as a template for other jurisdictions in developing their own ACT programs, thus allowing for differences in countries, regions and communities regarding mental health laws, policies and needed programs. The basic content of the standards has been validated through more than 20 years of field testing, though each version may differ to accommodate regional differences. Both the American standards and the Ontario standards documents are written in a narrative format, organizing them into several broad groups with sub-headings. Neither version clearly identifies individual standards nor provides a framework to facilitate evaluation of fidelity to standards.

### Survey Development

To present the revised standards in suitable survey format to assess participants' views regarding their ACT teams' level of fidelity to individual standards, the researchers were guided by the American standards document and earlier fidelity assessment tools; e.g., Test and Stein [35], presented guidelines for community treatment programs for the mentally ill; and Teague et al. [14], developed a list of 26 program criteria for fidelity to ACT. Additional input was sought from two ACT program content experts, both of whom had previously been ACT Program Coordinators and one had been involved in the development of the 2004 revised Ontario Program Standards for ACT Teams. This process resulted in the development of the *ACT Program Fidelity Tool^© ^*[15], which consisted of 33 discrete and equally weighted elements (statements of standards), specifically identifying and providing a short description for each of the standards described in the 2004 Ontario Program Standards for ACT Teams document.

The statements were used as the basis for the two key sets of assessment questions, each with 33 items (one item for each standard). Questions were designed to allow Program Coordinators to identify levels of compliance and importance based on their perceptions and intimate knowledge of the operation of his/her ACT program. The first set of questions assessed the extent to which participants perceive their ACT program to be in compliance with each of the 33 standards, and the second set of questions assessed the extent to which participants perceived each standard to be essential to effective functioning of the ACT program. Both sets of questions use 10-point Likert type scales, where "1" means "Not at all", and "10" means "Completely".

Of the 33 standards, the two which are most directly related to the research questions for this paper regarding participation by the community, are standards #32 (Community Advisory Bodies) and #25 (Peer Support Specialists). The standard regarding Community Advisory Bodies was paraphrased as follows in the survey:

*Each team shall have a Community Advisory Body which makes recommendations on the annual operating plan and budget, promotes fidelity to program standards, and reports directly to the Board of Directors of the sponsoring agency*.

The standard regarding the provision of Peer Support Services was paraphrased as:

*Each team shall provide peer support through the deployment of a peer specialist and the active participation of clients in service planning and development*.

### Scale Validity

Since the development of the two main measurement scales for the 33 program standards is based on results of the literature search, early ACT program fidelity tools [11], the American standards, the Ontario standards, and input from ACT Program Coordinators, the empirical measures should adequately represent the constructs being examined (i.e., compliance to and importance of the individual standards). Further, prior to its implementation the survey was pre-tested with four ACT Program Coordinators who reported that, in their expert opinions, the measurement scales "looked right" and the descriptions adequately represent the individual standards. Thus, verifying that content validity and face validity are high.

To assess criterion validity, we examine results using the *ACT Program Fidelity Tool*^© ^[15] in our study and compare them to similar measures for similar constructs by other researchers in other studies [36]. For example, McHugo et al. [13], using 5-point scales, report mean score ranges of fidelity to standards for assertive community treatment programs of 3.73-4.65, and for integrated dual disorders treatment scores of 2.71 - 4.21 (on a scale where 1 = no adherence to fidelity, and 5 = full adherence). This is generally consistent with the range of means resulting in our larger study where, using 10-point Likert-type scales, we found an average mean score of 8.73 (range of 5.68-9.98) for all standards, and a mean of 7.21 (range 2.44-8.21) for concurrent disorders (on a scale where 1 = not at all compliant, and 10 = completely compliant).

The final version of the survey consisted of four sections. Section A collected profile information about each of the ACT programs including: how long the program has been in existence; funding; and, information about the sponsoring organization and the services and benefits provided. Section B listed all 33 standards where respondents were given the following instruction: "On a scale of 1-10, where 1 means 'Not at all' and 10 means 'Completely', what is your opinion of the extent to which your ACT program meets this standard?", and if not in complete compliance, space was provided to record their response to the question, "What challenges prevent the Program from reaching a 10 on the scale?" Section C used a 10-point Likert-type scale to assess the extent to which respondents believed each of the 33 standards in the ACT Fidelity Tool is essential for effective functioning of their ACT Program (i.e., importance). And finally, Section D collected demographic data from each respondent, including professional and educational background, number of years as an ACT Program Coordinator and in total in the mental health field.

### Data Analysis

Quantitative data were entered into SPSS 16.0 and responses to open-ended questions were entered into Excel 2007 for further analysis. All statistical analyses were conducted at a 95% confidence level. Data analysis techniques for this paper included: frequencies and descriptives to create profiles of participating ACT teams and ACT Program Coordinators; independent t-tests to identify significant differences in means, for example between level of compliance versus level of importance; Pearson Correlations to identify significant correlations between variables and to examine construct validity; and, additional comparisons of sub-groups (e.g., rural versus urban) ACT teams.

A thematic approach to content analysis was used to code and analyze responses to all open-ended questions regarding barriers to compliance [37]. Three raters identified and established the major categories and sub-categories represented in respondents' comments regarding the challenges preventing complete compliance to each of the program standards. Agreement was reached on the final six major categories of barriers to full compliance: Human Resources, Communications, Client-Related, Organizational, Standard-Related, and External. For each major category of barrier, sub-codes were also identified and agreed upon. Each rater then independently coded the challenges identified by respondents into major categories and sub-codes. Initial inter-rater agreement was just over 90%. Following an iterative process of discussion and clarification among the raters, inter-rater reliability reached 100% on all coding of responses.

## Results

Program Coordinators from 56 of the 66 existing ACT programs (85% response rate) completed the telephone survey, each of which took from 45 minutes to an hour. Respondents were 75% female (n = 42), 25% male (n = 14). Non-respondents were evenly distributed geographically; 79% (n = 45) classified their ACT team as "urban", and 21% (n = 11) "rural". Respondents (ACT Program Coordinators) had from four to 33 years of experience in the mental health field (mean = 19.5; median = 20.0; SD = 6.45), and from four weeks to 17 years in their current position (mean = 4.2 years, median = 4.0, SD = 3.20). Fifty-one percent of respondents identified their professional background as nursing, 29% as social work, and 10% as occupational therapy.

In general, we found some operational variation across individual programs. This is not surprising since ACT programs are intended to meet the specific needs of the communities served [24]. No ACT program was identified by the Program Coordinators as being fully compliant with all 33 standards. However, compliance means of 9.0 or higher were reported for 16 of the standards, with means between 8.0 and 8.9 for 11 standards, and means of less than 8.0 for the remaining six standards (15). When asked to indicate the extent to which respondents feel that their program's sponsoring organization has supported and/or empowered their ACT program, the mean = 8.3, where 1 = "Not at all" and 10 = "Completely" (Median = 8.0, SD = 1.48). Fifty-four respondents answered this question, where the minimum value was "5" (n = 3), the maximum "10" (n = 17), and 14 answered "8".

### Community Advisory Bodies

The standard with the lowest perceived level of compliance (of all 33 standards) was the standard which requires each ACT program to have a Community Advisory Body (CAB), with a mean of 5.7 (median = 7.0, SD = 3.32). This same standard was also rated the least important of all the standards to the effective functioning of the ACT program with a mean of 6.0 (median = 6.5, SD = 2.62). Further, twelve of the Program Coordinators (25%) rated their ACT program's level of compliance with this standard as "1" (not at all compliant), while eight (14%) rated compliance as "10" (completely compliant).

Rural ACT teams are, on average, reported to be doing significantly better than their urban counterparts at complying with the standard for having a CAB, but they are still not in full compliance. The mean for rural programs is 7.5 (median = 8.0, SD = 2.46), while the mean for urban programs is 5.1 (median = 4.5; SD = 3.52). The difference in the means is significant (p = 0.047). Program Coordinators of rural programs also indicate they believe the requirement to have a CAB is more important than do the Program Coordinators of the urban teams (means of 6.3 versus 5.7 respectively), although this difference is not statistically significant.

The three major themes that emerged from the interviews with respect to the barriers to fully implementing the Community Advisory Body were: external issues; standard related issues; and, organizational/structural related issues. Fifty-one Program Coordinators provided a total of 165 reasons for lack of full compliance to this standard. These reasons for non-compliance range from simply "*Not getting around to it*" to *"it [CABs] doesn't work*". Table 1 presents a summary of coded responses, with sample comments regarding the barriers identified by respondents that prevent their ACT programs from being fully compliant with the standard for CABs.

**Table 1:** Summary of Barriers to Compliance with Standard for Community Advisory Body

Categories and Sub-categories	Number of Comments (% of total)	Selected Sample Comments (R = Respondent Id. No.)
**External Issues:** • Lack of support from sponsoring agency • Rely on external organization to meet standard	64 (39%)	• *Support from the sponsoring agency is required.(R14)* • *We need direction from the (sponsoring) agency to say we have to have one. Don't have one that's necessarily specific to the program.(R32)* • *There was a community advisory body, and that has been disbanded. Sponsoring agency is transitioning and the community advisory is falling to the wayside.(R45)* • *Host organization has a number of advisory bodies who all function in an advisory capacity for mental health services ... didn't create another just for ACT.(R60)* • *There have to be ways to meet the standards and there needs to be funding... we are losing our ability to do the rehab portion.(R30)* • *Advisory Board meets irregularly... lack of direction and goals.(R49)*

**Standard Related issues:** • Standard is not a top priority • Standard is unnecessary or unimportant • Standard should be modified	48 (29%)	• *The community advisory board was disbanded.(R1)* • *Hospital has one, not specific to ACT. I don't think we need one.(R17)* • *We have a committee... it is the bane of my existence. We struggled with their mandate... role of sharing information between teams... three teams share one advisory committee. It doesn't work.(RT52)* • *The standard is written for small community agencies, not large corporate hospitals.(R27)* • *The body became the Mental Health Advocacy Committee from the Consumer Advocacy Committee... not sure if it fits the mandate.(R16)*

**Organizational Issues:** • Process issues • Structural issues • Lack of motivation • Issues with unions	42 (25%)	• *Persons on community advisory boards are not permanent. Within a year they are off and doing something else.(R4)* • *Our community advisory group does not report to our hospital board but they are in communication with the Ministry of Health.(R64)* • *Advisory body meets irregularly...lack of direction and goals. Being reviewed and reassessed.(R49)* • *Not getting around to it.(R58)* • *There is no way my Community Advisory Body has the ear of the Board of Directors. The Board of Directors is not interested in that micro-level. They are more concerned with building million dollar wings. The Community Advisory Body sees the goals of the budget, but not the details. They don't have the authority to promote fidelity to the model. They're involved with advocacy issues.(R27)* • *Problems with quorum...lack of clear roles and responsibility for Advisory Board.(R42)*

**Other Issues;** • Client related, • Human resources • Communications	11 (7%)	• *Advisory body in place...information is not provided for which they could provide full advisory function. (R47)* • *Commitment required from staff to meet the standards. Need to look at teams, geography, and what they are doing. Some clients don't want to be seen as often or be involved in planning.(R36)*

TOTAL	165 (100%)	

### Peer Support

The standard regarding the provision of peer support services through the use of a Peer Support Specialist received the second lowest compliance rating of the 33 standards, with a mean of 6.2 (median = 8.0, SD = 3.74). Sixteen of the Program Coordinators (29%) rated their ACT program's level of compliance with this standard as "1" (not at all compliant). However, when asked to indicate how essential this standard was to the effective functioning of the ACT Program, it was rated much higher (mean of 8.1; median = 8.0, SD = 2.16). The difference between the means for level of compliance and how essential respondents felt the standard to be was highly significant (*t(_47_) *= *p *< .001).

Urban ACT programs (n = 45) are doing slightly better than their rural (n = 11) counterparts at complying with this standard, although on average neither group is fully compliant. Specifically, the mean on the 10-point scale for urban ACT programs was 6.2 (median = 8.0, SD = 3.76), while the mean for rural programs was 5.2 (median = 5.0, SD = 3.68). The difference in means is not statistically significant. However, the rural Program Coordinators felt this standard was slightly more important to the functioning of the program than did the urban Program Coordinators, with means of 8.7 and 7.8 respectively (difference not statistically significant).

The three major themes that emerged from the interviews with respect to the barriers to fully implementing the Peer Support Specialist role were: human resource related issues; organizational/structural related issues; and, standard related issues. Forty-seven respondents provided a total of 73 comments regarding barriers to full compliance with the standard for Peer Support Specialists. The reasons provided for non-compliance range from "*we are resolving union issues*" to "*having trouble recruiting*". Table 2 provides a summary of the coding and analysis of the reasons for not having a Peer Support Specialist on their ACT program team, and includes sample quotes from respondents to exemplify the concerns expressed within each of the major coded categories.

**Table 2:** Summary of Barriers to Compliance with Standard for Peer Support Specialist

Categories and Sub-categories	Number of Comments (% of total)	Selected Sample Comments (R = Respondent Id. No.)
**Human Resource Issues:** • Staff shortages (recruiting difficulties, maternity leave, illness) • Staff training required	35 (48%)	• *Peer support worker requires clear job description and adapted (role). Should be a mental health worker first and a peer specialist second. If they are going to carry primes, they need to have the skills. It doesn't work if skills are less.(R20)* • *A peer is someone who once met the admission criteria, lived the experience, and who can also function in a meaningful way... difficult to find.(R61)* • *Having trouble recruiting the position.(R4)* • *Seems to take a long time for a peer support worker to get into the role. They don't go to school for the peer support role--once they get there it is fabulous.(R41)* • *[Our] peer support specialist is on sick leave for approximately one year.(R49)*

**Standard Related Issues:** • Standard deemed unattainable/unrealistic/unnecessary/unimportant • Standard needs improvement/modification • Standard requires clarification (or not aware of standard)	21 (29%)	• *The definition of a peer was stretched a little bit... [Our] peer doesn't have a major mental illness.(R30)* • *The peer support specialist is excellent ... just ill enough to be a peer support. Need to back up and support if the peer support needs time off. Question whether there should be a different standard... from general to evaluation. The work that this person does is a "10"... contributes a lot... does leisure and recreation.(R31)* • *Lack of clarity of the role... what added value does it bring?(R60)* • *Peer support is only .7 FTE ... cannot take a full patient load. Full caseload is not possible.(R46)*

**Organizational Issues:** • Structural barriers, e.g. problems with ODSP* and/or unions • Standard implementation is a "work in progress" • Process barriers *Ontario Disability Support Program	17 (23%)	• *The intention is to have a peer specialist... we are resolving union issues.(R23)* • *We had a peer support working in the past (for about a year and a half) but no longer have the position due to demands of supervising this person and union dispute over the classification of the job. (e.g., which bargaining unit should it fall under? But we do acknowledge that the position is needed.(R8)* • *Probably other teams hire from a different mental health population.(R61)* • *We don't have the position in a formal way. Will recruit ... once a position is freed up. This is a new standard.(R19)*

TOTAL	73 (100%)	

Pearson correlations were used to examine the association between the perceived compliance with, and the perceived level of importance of, the two community participation standards (Table 3). The four variables were positively and significantly correlated with one another. Specifically, moderate, positive correlations were found between level of compliance with having a Peer Support Specialist and level of compliance with having a CAB and, level of compliance with having a Peer Support Specialist and level of importance of the CAB.

**Table 3:** Pearson Correlations between Importance and Compliance for Community Participation Standards

	Compliance: Peer Support Specialist	Compliance: Community Advisory Body	Importance: Peer Support Specialist	Importance: Community Advisory Body
**Compliance: Peer Support Specialist**	1	.346*	.596**	.323*

**Compliance: Community Advisory Body**	.346*	1	.355*	.453**

**Importance: Peer Support Specialist**	.596**	.355*	1	.566*

**Importance: Community Advisory Body**	.323*	.453**	.556**	1

* Correlation is significant at the 0.05 level (2-tailed)

** Correlation is significant at the 0.01 level (2-tailed)

Table 3 also presents our findings of positive correlations between: the level of compliance with and the perceived importance of having a Peer Support Specialist; the level of compliance with and the level of importance of having a CAB; and, the perceived importance of having a Peer Support Specialist and the level of importance associated with having a CAB.

Additionally, a positive and significant correlation was found between perceived sponsoring organization support and the level of importance ascribed to having a CAB (*r *= .36, *p *< .05).

Finally, significant and positive correlations were found between level of compliance with the standard to have a CAB and with each of the standards related to the provision of: crisis assessment and intervention, 24 hours a day, seven days a week (*r *= .44, *p *< .001); concurrent mental health and addiction disorder services (*r *= .30, *p *< .05); social/interpersonal and leisure skill training (*r *= .29, *p *< .05); family-centred services, including education, conflict resolution, and related support (*r *= .33, *p *< .05); and, performance improvement and program evaluation which includes criteria and methods for assessing client outcomes, client and family satisfaction, and fidelity to the ACT model (*r *= .33, *p *< .05).

## Discussion

In our analysis, we examine in detail the extent to which ACT programs are complying with standards that relate to involving the community's participation in the ACT Program and the extent to which these standards are perceived as important; specifically, the requirements to have a Community Advisory Body (CAB) composed of representatives from the immediate community, and a Peer Support Specialist as a member of each ACT Team. Despite efforts to implement evidence-based ACT program standards, there remains substantial variation in the extent to which individual programs comply with both of these community participation standards, a finding consistent with past research on ACT programs in other jurisdictions [29]. In this study we found that barriers to compliance with standards for community participation are primarily related to external issues, concerns about the standard itself, and/or organizational problems. A large number of ACT programs have no CAB in place, or they have one in theory but it simply does not meet. As well, many ACT programs have yet to fill their required Peer Support Specialist position, citing reasons such as: difficulty in finding candidates who fit the criteria and who are also functioning at a high enough level to assume the position; and, lack of clarity of the duties of the peer support specialist. These findings are also consistent with prior research on the use of Peer Support Specialists [38,39]. It is interesting to note the significant and positive correlations between compliance with having a CAB and the compliance with provision of several services that are essential to the improved well-being of the programs' clients. Further research should be conducted to investigate this possible positive influence of having a CAB on fidelity to other program standards.

While our results revealed a wide range of reasons provided for ACT teams failing to fully comply with the two standards requiring the community's participation (as categorized in Table 1 and Table 2), our analysis of comments also reveals two important issues that contribute to lower levels of compliance that cut across our main categories. These are: (1) internal resistance (within the ACT programs themselves) to implementation; and, (2) the lack of sponsoring agency support (or even active opposition by some sponsoring agencies to implementation).

### Internal Resistance

The Program Coordinators were generally supportive of CABs, with several indicating that the CAB was an extremely valuable resource which enhanced decisions related to service delivery. However, in a few instances the Program Coordinators seemed to feel that an advisory body was essentially a waste of time. These individuals espoused a rather paternalistic view of health care in which the health professionals are believed to be more capable of making decisions than either consumers or members of the broader community. This is somewhat surprising given that the philosophy at the core of the ACT movement is to empower consumers and achieve integration with the community. It also suggests that some Program Coordinators are either unaware of the research evidence regarding the positive value of CABs, or they may be unable to accept research findings that may be inconsistent with their personal beliefs and values.

The findings in relation to the use of Peer Support Specialists had some similarities to those regarding CABs. Most Program Coordinators praised the benefits these individuals brought to their organizations, while a smaller number questioned the value of having non-professionals in a service delivery role. An additional problem with meeting this particular standard was finding the right fit of an individual who had been a consumer of mental health services but is now sufficiently stable to fill this rather demanding position. To some extent, this may be beyond the control of the individual ACT programs. However, Program Coordinators who were strongly in favour of using Peer Support Specialists seemed to do better in overcoming this obstacle than those that were less supportive. This assessment is further supported by the positive and statistically significant correlations between Program Coordinators' perceived level of compliance and their perceived level of importance for the two community participation standards.

One possible explanation for the variation in views from the Program Coordinators may lie in their professional orientation. In general, Program Coordinators from a nursing background were less likely to support the need for either CABs or peer specialists, perhaps feeling comfortable that they had sufficient knowledge and experience to provide similar perspectives and approaches to that which the CAB and/or Peer Support Specialist would provide. This is an area where additional research would be helpful.

### Lack of Sponsoring Agency Support

An unexpected finding was the reported degree to which some sponsoring agencies resisted, or refused to allow, CABs to be established; and further, if they were established, prevented the CABs from reporting directly to the Boards of Directors of the sponsoring agencies. This was surprising given the specific expectations set out in the revised standards for the sponsoring agencies to support the implementation of program standards, explicitly requiring that a mechanism be in place for CABs to report directly to their Boards. However, this behaviour is consistent with the findings of other researchers who comment that there exists "... a generally unfavourable policy environment and hospital institutional culture that poses significant barriers..." to such collaborations [40].

In our study, in some instances where one sponsoring agency was responsible for more than one ACT program, there was resistance to forming separate CABs for each program. Instead the sponsoring agencies often simplified the process by forming a single body to serve in an advisory capacity for all ACT programs under their supervision. However, in a large number of cases it appears that the sponsoring agencies simply felt that there was either no need for a CAB or they were concerned that a volunteer advisory body might interfere in some way with their decision-making processes or overall governance. This assessment is further supported by the positive and statistically significant correlation between the extent to which Program Coordinators' perceived sponsoring agencies as being supportive and their perceived level of implementation of the CAB standard. That is, higher (lower) perceived support from the sponsoring agency was correlated with higher (lower) perceived compliance to the standard.

Sponsoring agency support (or lack of support) was much less of an issue when it came to the use of Peer Support Specialists. Opposition tended to be more passive as some ACT programs were left to set their own staffing priorities. Not surprisingly, Peer Support Specialists seemed to be more expendable than other health care providers when staffing decisions had to be made.

The major limitation to this study is that we collected data only from the perspective of the ACT Program Coordinators. It would be advantageous to have data from an independent source (outside audit) to compare results with those of the Program Coordinators. There was, however, very limited availability of such information. Data representing the views of other ACT team members would also provide useful additional information. Another potential problem, since this was a telephone survey asking respondents to provide ratings and comments regarding a wide variety of program activities, was that of inaccurate recall on the part of respondents. We minimized this potential problem by (1) sending all respondents a hard copy of the survey document prior to the actual telephone interview, and (2) calling prior to the survey to see if they had questions or needed clarification about any of the survey items. It was anticipated that social desirability bias might introduce error to the study, but since there was such wide dispersion of responses to each of the rating questions (compliance and importance), it seems that this was not a problem.

## Conclusion

Regardless of the country or regional jurisdiction which is using the ACT model, the results of this study point to the need for identifying barriers to the implementation of community participation program standards if implementation is to be successful and the delivery of these programs is to be evidence-based. Given that the resistance to implementing standards related to the inclusion of Community Advisory Bodies and Peer Support Specialists is both internal to the ACT programs and within the sponsoring agencies, an effort to address both areas is required. One possible approach to improving implementation of community participation standards may lie in improving the education of ACT program administrators and team members about the evidence supporting the use of the various standards [41]. While education alone will not overcome the structural barriers identified, it should bring about greater awareness of the importance of complying with the standards. Another approach might be to enhance the clarity of the standards and to implement a proactive approach to monitoring compliance [42]. The implication for administrators and policy makers of failing to do so is that some of Ontario's ACT programs will not achieve the degree of success, improved patient outcomes, and increased cost effectiveness, attained by other programs around the world, and scarce mental health resources will have been used in a less than ideal manner.

## Competing interests

The authors declare that they have no competing interests.
